# Prevalence of Alcohol in Unintentional Opioid Overdose Deaths, 2017-2020

**DOI:** 10.1001/jamanetworkopen.2022.52585

**Published:** 2023-01-24

**Authors:** Aryn Z. Phillips, Lori A. Post, Maryann Mason

**Affiliations:** 1 Department of Preventive Medicine, Feinberg School of Medicine, Northwestern University, Chicago, Illinois; 2 Department of Health Policy and Management, University of Maryland School of Public Health, College Park; 3 Buehler Center for Health Policy and Economics, Feinberg School of Medicine, Northwestern University, Chicago, Illinois

## Abstract

This cross-sectional study investigates the prevalence of alcohol in unintentional opioid overdose deaths in Illinois from 2017 through 2020.

## Introduction

Although there is substantial literature on opioid overdose deaths (OODs) and polysubstance use, involvement of alcohol in these deaths is less clear. The “fourth wave” of the opioid crisis (2019-present) and the overlapping COVID-19 pandemic likely affected alcohol use among individuals who use opioids. The fourth wave of the opioid crisis is characterized by opioid-stimulant polysubstance use^1^; stimulants can mask alcohol’s sedative effects,^2^ perhaps resulting in greater alcohol consumption. The COVID-19 pandemic has also amplified risks for increased alcohol use (eg, reduced access to substance use treatment services^3^ and increased distress^4^). However, it is not evident whether these events have translated into changes in the substances involved in OODs.

We investigated the prevalence of alcohol in OODs from July 1, 2017, to December 31, 2020, in Illinois, including temporal associations between Illinois’ COVID-19 stay-at-home order (March 21 to June 5, 2020) and the prevalence of alcohol in OODs. We also assessed differences by sex, race, ethnicity, and age.

## Methods

Data on unintentional OODs were culled from the Illinois State Unintentional Drug Overdose Reporting System (SUDORS). After excluding records without results of alcohol toxicology tests (n = 685) or those missing data on decedents’ sex, race, ethnicity, or age (n = 1), the analytic sample contained 6774 OOD records. The study was deemed exempt from review by the Northwestern University institutional review board because the data are deidentified and the research is on deceased individuals, which is not considered human participants research and not required to undergo review. This study followed the STROBE reporting guideline.

Alcohol involvement was assessed using toxicology reports, which describe positive findings of ethanol in peripheral blood, urine, or liver tissue (a less-common but suitable specimen for detection). Deaths were classified as involving alcohol if any level of ethanol was detected.

Statistical analysis was conducted from October 5, 2021, to March 10, 2022. We used a modified Poisson interrupted time series model to assess the role of alcohol in OODs and any changes that occurred during and after the stay-at-home order. Differential trends by sex (male or female), race and ethnicity (analyses by race and ethnicity were limited to those who were non-Hispanic White, non-Hispanic Black, and Hispanic due to the limited number of those of other race and ethnicity [all decedents who were not identified as Hispanic, non-Hispanic Black, or non-Hispanic White; n = 61]), and age (≥55 or <55 years) were investigated using interaction terms. All *P* values were from 2-sided tests, and results were deemed statistically significant at *P* < .05. Analyses were conducted in Stata, version 17 (StataCorp LLC). Further details are available in the eAppendix in Supplement 1.

## Results

Of 6774 total OODs (5033 males and 1741 females; 5233 aged <55 years and 1541 aged ≥55 years), alcohol was involved in 2073 OODs (30.6%). Decedent demographic characteristics are presented in Table 1. The model failed to identify any significant trend over time prior to the COVID-19 stay-at-home order nor any significant immediate changes in prevalence level or changes in trends after the implementation or withdrawal of the stay-at-home order (Table 2). The models that incorporated sex, race, ethnicity, and age revealed differences in alcohol involvement in the years prior to the order. The prevalence of alcohol use in OODs was higher for men than women (prevalence ratio [PR] for women, 0.51; 95% CI, 0.40-0.66), for non-Hispanic Black and Hispanic decedents than White decedents (non-Hispanic Black decedents: PR, 1.71; 95% CI, 1.39-2.09; Hispanic decedents: PR, 1.59; 95% CI, 1.22-2.07), and for decedents aged 55 years or older than decedents younger than 55 years (aged ≥55 years: PR, 1.38; 95% CI, 1.13-1.68). There were no significant changes by subgroup associated with COVID-19 policy changes.

**Table 1.** Total Unintentional Opioid Overdose Deaths and Prevalence of Alcohol Involvement in Illinois, July 2017 to December 2020

Characteristic	Overall deaths		Deaths before SAH		Deaths during SAH			Deaths after SAH	
	Total, No.	Alcohol involved, No. (%)	Total, No.	Alcohol involved, No. (%)	Total, No.	Alcohol involved, No. (%)	Total, No.		Alcohol involved, No. (%)
Total	6774	2073 (30.6)	4865	1501 (30.9)	591	155 (26.2)	1318		417 (31.6)
Sex									
Female	1741	379 (21.8)	1275	277 (21.7)	151	26 (17.2)	315		76 (24.1)
Male	5033	1694 (33.7)	3590	1224 (34.1)	440	129 (29.3)	1003		341 (34.0)
Race and ethnicity									
Hispanic	765	298 (39.0)	517	210 (40.6)	73	24 (32.9)	175		64 (36.6)
Non-Hispanic									
Black	2147	711 (33.1)	1462	492 (33.7)	210	66 (31.4)	475		153 (32.2)
White	3568	960 (26.9)	2627	711 (27.1)	294	60 (20.4)	647		189 (29.2)
Other^a^	294	104 (35.4)	259	88 (34.0)	14	5 (35.7)	21		11 (52.4)
Age, y									
<55	5233	1560 (29.8)	3792	1146 (30.2)	450	118 (26.2)	991		296 (29.9)
≥55	1541	513 (33.3)	1073	355 (33.1)	141	37 (26.2)	327		121 (37.0)

Abbreviation: SAH, stay-at-home order (from March 21 to June 5, 2020).

^a^ Includes all decedents who are not identified as Hispanic, non-Hispanic Black, or non-Hispanic White.

**Table 2.** Alcohol Involvement in Unintentional Opioid Overdose Deaths in Illinois, July 2017 to December 2020 (N = 6774)

Variable	Prevalence ratio (95% CI)^a^
Time to date of death	0.999 (0.999-1.000)
SAH	
Before	1 [Reference]
During	0.161 (0.000-172.403)
After	0.293 (0.047-1.819)
Time × during SAH	1.002 (0.995-1.008)
Time × after SAH	1.001 (0.999-1.003)

Abbreviation: SAH, stay-at-home order (from March 21 to June 5, 2020).

^a^ From a modified Poisson regression with robust SEs and adjusted for seasonality.

## Discussion

Over 30% of unintentional OODs in Illinois from July 2017 to December 2020 involved alcohol. There was no evidence of changes in the prevalence of alcohol in unintentional OODs during or after the COVID-19 stay-at-home order. It is possible our sample size was too small or our period under study too short to detect an association; additional studies are needed, including those that can identify cause if these findings are corroborated. Nonetheless, the fact that 30% of OODs involved alcohol suggests that prevention and mitigation strategies should address alcohol use. Specific attention is warranted given that alcohol may be viewed differently than illicit substances by individuals who use opioids and by treating clinicians^5^ and given alcohol’s detrimental effect on the liver and the high rates of hepatitis C among individuals who inject drugs.^6^

Limitations of this study include crude measurements of intent (ie, SUDORS may miss the nuances of quasi-suicidal deaths in which decedents feel ambivalent toward life and death) and the extent of alcohol use (ie, we could not determine how much alcohol was consumed).

## Notes

Supplement 1.
eAppendix.

eReferences.

Supplement 2.
Data Sharing Statement
