# Supplementary material for: Prevalence of Alcohol in Unintentional Opioid Overdose Deaths, 2017-2020
Source: JAMA Netw Open. 2023 Jan 24;6(1):e2252585. doi: 10.1001/jamanetworkopen.2022.52585 (PMC10408257; doi:10.1001/jamanetworkopen.2022.52585)
Supplement: Supplement 1. — eAppendix. eReferences. [file jamanetwopen-e2252585-s001.pdf]

Supplementary Online Content

Phillips AZ, Post LA, Mason M. Prevalence of alcohol in unintentional opioid overdose deaths, 2017-2020. *JAMA Netw Open.* 2023;6(1):e2252585. doi:10.1001/jamanetworkopen.2022.52585

eAppendix.

eReferences.

This supplementary material has been provided by the authors to give readers additional information about their work.

© 2023 Phillips AZ et al. *JAMA Network Open.*

eAppendix

Data

The data are from the Illinois Statewide Unintentional Drug Overdose Reporting System (SUDORS).[1] SUDORS is a database of unintentional overdose fatalities, information about which is abstracted from death certificates, coroner/medical examiner reports, toxicology reports, and autopsies. It is funded as part of the Centers for Disease Control and Prevention's Enhanced State Opioid Overdose Surveillance (ESOOS), which supports states in collecting timely and high-quality data on drug overdose events and their risk factors with the goal of informing prevention and response efforts.[2] SUDORS has been collecting data on unintentional overdose deaths in Illinois since 2017. Counties participate voluntarily, and the database includes over 86 percent of the overdose deaths occurring in the state. The study was exempt from review by the institutional review board.

Measures

Alcohol involvement was determined using toxicology reports, which report positive findings of ethanol in peripheral blood, urine, or liver tissue. While blood and urine are the most common specimens for ethanol testing, liver tissue can also be used, particularly when blood and urine are not available. There may be some continued metabolism of alcohol in the liver postmortem, but detection of ethanol in liver tissue still signifies ethanol's presence at time of death.[3] Deaths were classified as involving alcohol if any level of ethanol was detected, and involvement was measured using a binary variable for which decedents were assigned a value of 1 if alcohol was involved and a value of 0 if not.

© 2023 Phillips AZ et al. *JAMA Network Open.*

Date of death was documented using the calendar date (day, month, year) and operationalized using a variable for which values represent the days elapsed between the start of the study period (July 1, 2017) and the date of death. Sex was measured using biological sex with possible values of male and female. Race and ethnicity were operationalized using a categorical variable with mutually exclusive categories for non-Hispanic White, non-Hispanic Black, Hispanic, and other race or ethnicity. Age was measured using a binary variable for which an individual received a value of 0 if they were less than 55 years old at the time of death and 1 if 55 years or older. Age of 55 was chosen to best correspond to the ages of the baby boomer generation, among whom alcohol use and misuse have been increasing at a rapid rate.[4,5]

Statistical analysis

We used interrupted time series models[6–8] to assess trends in alcohol involvement in OODs and any changes that occurred during and following the stay-at-home order. These models regressed alcohol involvement on time from start of the study period to date of death, a binary indicator for the death occurring during the stay-at-home order (1) or before (0), and their interaction, as well as a binary indicator for the death occurring after the stay-at-home order (1) or before (0) and its interaction with time to date of death. In this model, the coefficient of the time to death term represents the trend (i.e. slope) in prevalence of alcohol involvement over time prior to the stay-at-home order. The coefficient of the indicator for the death taking place during the stay-at-home order represents an immediate change in prevalence of alcohol involvement (i.e. level) following the implementation of the order compared to before and coefficient of

© 2023 Phillips AZ et al. *JAMA Network Open.*

the indicator for after the stay-at-home indicator represents a change in prevalence following the withdrawal of the order compared to before the order. The interactions represent the change in the trend following the implementation or withdrawal of the order relative to the trend before the order. The model was estimated using modified Poisson regression with robust standard errors[9] and adjusted for seasonality using Fourier terms. This adjustment involves including pairs of sine and cosine functions of time, which model seasonal variation in regular waves, in the regression equation.[7,10]

We then investigated differential trends and impacts of the stay-at-home order by sex, race and ethnicity, and age by estimating separate models for each of these characteristics. These models additionally included a main effect of the characteristic (e.g. female versus male), its interaction with time to date of death, its interaction with each of the stay-at-home order indicators, and triple interactions between the characteristic, time, and stay-at-home order indicators. The analysis for race and ethnicity was limited to decedents who were non-Hispanic White, non-Hispanic Black, or Hispanic due to very limited representation of other races (n=61).

Estimates were considered statistically significant if p values were less than 0.05. All analyses were conducted in Stata version 17.

© 2023 Phillips AZ et al. *JAMA Network Open.*

eReferences

1. Northwestern Feinberg School of Medicine, Buehler Center of Health Policy & Economics. *Statewide Unintentional Drug Overdose Reporting System (SUDORS)*. https://sites.northwestern.edu/illinoissudors/

2. Centers for Disease Control and Prevention, National Center for Injury Prevention and Control. *Enhanced State Opioid Overdose Surveillance*.; 2020. https://www.cdc.gov/drugoverdose/foa/state-opioid-mm.html

3. Kugelberg FC, Jones AW. Interpreting results of ethanol analysis in postmortem specimens: A review of the literature. *Forensic Sci Int*. 2007;165(1):10-29. doi:10.1016/j.forsciint.2006.05.004

4. Grucza RA, Sher KJ, Kerr WC, et al. Trends in adult alcohol use and binge drinking in the early 21st-Century United States: A Meta-Analysis of 6 National Survey Series. *Alcohol Clin Exp Res*. 2018;42(10):1939-1950. doi:10.1111/acer.13859

5. Barry KL, Blow FC. Drinking over the lifespan: Focus on older adults. *Alcohol Res Curr Rev*. 2016;38(1):115-120.

6. Linden A. Conducting interrupted time-series analysis for single-and multiple-group comparisons. *Stata J*. 2015;15(2):480-500.

7. Lopez Bernal J, Cummins S, Gasparrini A. Interrupted time series regression for the evaluation of public health interventions: a tutorial. *Int J Epidemiol*. Published online June 9, 2016:dyw098. doi:10.1093/ije/dyw098

8. Beard E, Marsden J, Brown J, et al. Understanding and using time series analyses in addiction research. *Addiction*. 2019;114(10):1866-1884. doi:10.1111/add.14643

9. Zou G. A modified poisson regression approach to prospective studies with binary data. *Am J Epidemiol*. 2004;159(7):702-706. doi:10.1093/aje/kwh090

10. Bhaskaran K, Gasparrini A, Hajat S, Smeeth L, Armstrong B. Time series regression studies in environmental epidemiology. *Int J Epidemiol*. 2013;42(4):1187-1195. doi:10.1093/ije/dyt092

© 2023 Phillips AZ et al. *JAMA Network Open.*
