# Supplementary material for: Prevalence of Alcohol in Unintentional Opioid Overdose Deaths, 2017-2020
Source: JAMA Netw Open. 2023 Jan 24;6(1):e2252585. doi: 10.1001/jamanetworkopen.2022.52585 (PMC10408257; doi:10.1001/jamanetworkopen.2022.52585)
Supplement: Supplement 2. — Data Sharing Statement [file jamanetwopen-e2252585-s002.pdf]

Data Sharing Statement

Phillips. Prevalence of Alcohol in Unintentional Opioid Overdose Deaths, 2017-2020. *JAMA Netw Open*. Published January 24, 2023. doi:10.1001/jamanetworkopen.2022.52585

Data
Data available: No

Additional Information
Explanation for why data not available: The data are subject to a data use agreement. Interested parties should contact the corresponding author.
